# Lung cancer diagnosis based on weighted convolutional neural network using gene data expression

**DOI:** 10.1038/s41598-024-54124-7

**Published:** 2024-02-13

**Authors:** Thangamani M, Manjula Sanjay Koti, Nagashree B.A, Geetha V, Shreyas K.P, Sandeep Kumar Mathivanan, Gemmachis Teshite Dalu

**Affiliations:** 1 Department of Computer Science and Engineering, Hindusthan Institute of Technology, Valley Campus, Pollachi Highway, Othakkalmandapam (Post), Coimbatore, Tamil Nadu 641032 India; 2 Department of Master of Computer Applications, Dayananda Sagar Academy of Technology and Management, Bangalore, Karnataka 560082 India; 3 Department of Computer Science, School of Computing, Amrita Vishwa Vidyapeetham, Mysuru, 570026 India; 4 Department of Computer Science, School of Applied Sciences, REVA University, Bangalore, 560064 India; 5 Department of Computer Science and Applications, School of Computer Science and Applications, REVA University, Bangalore, 560064 India; 6 School of Computing Science and Engineering, Galgotias University, Greater Noida, 203201 India; 7 Department of Software Engineering, College of Computing and Informatics, Haramaya University, POB 138 Dire Dawa, Ethiopia

**Keywords:** Lung cancer, Health care, Medical research

## Abstract

Lung cancer is thought to be a genetic disease with a variety of unknown origins. Globocan2020 report tells in 2020 new cancer cases identified was 19.3 million and nearly 10.0 million died owed to cancer. GLOBOCAN envisages that the cancer cases will raised to 28.4 million in 2040. This charge is superior to the combined rates of the former generally prevalent malignancies, like breast, colorectal, and prostate cancers. For attribute selection in previous work, the information gain model was applied. Then, for lung cancer prediction, multilayer perceptron, random subspace, and sequential minimal optimization (SMO) are used. However, the total number of parameters in a multilayer perceptron can become extremely large. This is inefficient because of the duplication in such high dimensions, and SMO can become ineffective due to its calculating method and maintaining a single threshold value for prediction. To avoid these difficulties, our research presented a novel technique including Z-score normalization, levy flight cuckoo search optimization, and a weighted convolutional neural network for predicting lung cancer. This result findings show that the proposed technique is effective in precision, recall, and accuracy for the Kent Ridge Bio-Medical Dataset Repository.

## Introduction

### Lung cancer

Lung cancer is a deadly disease in both men and women. However, its prediction remains quite dismal, with a five-year survival rate of around 10% across most international locations. As a result, anticipating lung cancer at an early stage is critical to provide proper therapy to the patient and extend their life expectancy. Microarray technology is very effective at detecting and visualizing various cancer types. Cancer categorization is the most promising use of this technique, and it has received substantial research globally^1,2^. Because traditional diagnostic procedures depend on the subjective judgment of the morphological emergence of the tissue test, it is challenging to classify tumors with similar histopathological appearance (phenotype). However, with the growth of microarray technologies, analysts applied expression array analysis in their investigations. The rationale for this is that the microarray records the activity of a few thousand of genes simultaneously and few genetics are pertinent to illness^3–5^.

### Microarray technology

Microarray is an excellent tool for diagnosis and precise class prediction. Microarray technology is pivotal in lung cancer classification, providing a comprehensive snapshot of gene expression profiles. This high-throughput method allows simultaneous analysis of thousands of genes, offering unparalleled insights into molecular signatures. By measuring the abundance of mRNA transcripts, microarrays illuminate the intricate genetic landscape of lung cancer. The relevance of microarrays lies in their ability to distinguish between normal and cancerous tissues based on gene expression patterns. This technology facilitates the identification of specific genes associated with lung cancer subtypes, aiding in precise classification. Moreover, microarrays contribute to the discovery of biomarkers indicative of disease progression, prognosis, and potential therapeutic targets.

Proposed our research leverages microarray technology for cancer categorization, a subject of substantial global research interest. This demonstrates the effectiveness of utilizing advanced molecular techniques for precise and nuanced cancer classification, contributing to the broader scientific discourse on innovative methodologies in cancer research.

### Levy flight cuckoo search optimization algorithm

The Levy Flight Cuckoo Search combines Levy flights (LFCS) and cuckoo behaviour for efficient optimization, enhancing exploration–exploitation balance in search space. The working function as follows. *Initialization*: Potential solutions are represented as nests, each corresponding to a set of features (genes). Eggs (solutions) are laid in nests randomly. *Fitness Evaluation*: The fitness of each solution is assessed based on an objective function, often related to the optimization problem (e.g., maximizing prediction accuracy). *Levy Flights*: Inspired by Levy flights observed in nature, some cuckoos replace their eggs with new ones by performing a Levy flight. This involves moving in a series of steps with step lengths generated from Levy distribution. *Egg Laying*: Solutions with higher fitness replace the less fit ones in the nests, imitating the reproductive strategy of cuckoos. This is guided by the optimization goal. The process iterates, with cuckoos adapting their positions through Levy flights and replacing less fit solutions until convergence.

The novel incorporation of the LFCS algorithm for gene selection in lung cancer ordering holds promise for enhancing diagnostic accuracy. This innovative approach signifies a potential leap forward in refining the precision of lung cancer diagnostics.

There has been a lot of research done on lung cancer classification. However, there are still some accuracy difficulties. To address these concerns, the existing work information gain model was used to select attributes. Then, for lung cancer prediction, multilayer perceptron (MLP), random subspace, and SMO are used^6,7^. However, the total number of parameters in a MLP can become extremely large. This is ineffective because of the duplication in such high dimensions, and SMO can become unreliable due to its calculating method and retaining a single threshold value for prediction. To overcome these difficulties, our study proposed a new technique for predicting lung cancer. First, the scale of the input values will be normalized using z score normalization as the first step. Then, using the levy flight cuckoo search optimization algorithm, important genes will be chosen. Finally, a weighted CNN will be utilized to predict lung cancer.

Lung cancer is identified as a genetic disease with unknown origins, emphasizing the need for understanding the genetic factors contributing to the disease. The proposed technique addresses the inefficiencies associated with large parameter counts in MLP’s and potential ineffectiveness of SMO, contributing to more efficient and accurate lung cancer prediction. The research specifically applies and evaluates the proposed technique on the Kent Ridge Bio-Medical Dataset Repository, providing a concrete context for the study and demonstrating the practical implications of the findings. The research specifically applies and evaluates the proposed technique on the Kent Ridge Bio-Medical Dataset Repository, providing a concrete context for the study and demonstrating the practical implications of the findings.

The motivation for a novel lung cancer prediction technique stemmed from the limitations of existing methods. High parameter counts in MLP’s and inefficiencies in SMO were identified. These challenges led to suboptimal accuracy and computational inefficiency. The novel technique aims to address these issues by proposing a hybrid model, optimizing predictive accuracy, and overcoming the drawbacks of traditional approaches in lung cancer prediction.

Literature reviews given in section "Related work", section "Proposed system" organized as proposed system architecture and implementation steps. The section "Experimental outcome" indicates the result, discussion and interpretation of the result. Practical advantage and Limitation of the proposed research is presented in Section five and Section six for conclusion and future work.

## Related work

In previous lung cancer work, the information gain model was applied to identify and prioritize significant features for predictive modelling. By assessing the information gain of each attribute, the model helped select the most informative features, optimizing the performance of subsequent machine learning (ML) algorithms, such as MLP, random subspace, and SMO, in lung cancer prediction.

Alanni et al.^8^ designed a novel feature extraction strategy. The Gain Ratio (GR) and Improved Gene Expression Programming (IGEP) algorithms are used in gene screening and attribute extraction. The suggested method was evaluated using eight microarray datasets recorded using the eave-one-out cross-validation (LOOCV) method and Support Vector Machine (SVM) compared to other current feature selection methodologies, the model results reveal the effectiveness of the suggested strategy in choosing a minimal number of features while providing improved categorization accuracies.

Zhang et al.^9^ described svm based on Recursive Feature Elimination and Parameter Optimization (SVM-RFE-PO). In the attribute selection phase, the grid search (GS) approach, the Particle Swarm Optimization (PSO) algorithm, and the genetic algorithm (GA) are used to find the best parameters and the new attribute selection method includes, SVM-RFE-GS, SVM-RFE-PSO, and SVM-RFE-GA respectively. The best attribute subsets are then helped to prepare the SVM classifier for cancer classification. Random forest feature selection (RFFS), random forest feature selection and grid search (RFFS-GS), and the minimum Redundancy Maximum Relevance (mRMR) technique are employed for attribute extraction. The results showed that SVM-RFE-PSO method outperformed the testing data set in terms of Area under Curve (AUC). This approach not only saves time but also extracts more representative and functional genes.

Peng et al.^10^ presented a novel approach termed Discriminant Projection Shared Dictionary Learning (DPSDL). The technique creates a pooled vocabulary, drive in Fisher discriminant criteria to create a class-explicit sub-vocabulary, and calculates sign coefficients. Simultaneously, a projection matrix is skilled to enlarge the gap among samples. Test findings suggest that this approach outperforms current techniques for classification based on gene expression profiles. Thangamani et al.^11–13^ used ML approaches for disease prediction in medical applications.

Hu^14^ investigates the method called SE-Net for image classification with different datasets. It describes the various features of network strategy and association between channels. The authors make feature recalibration and reduce overwhelm features and produce relevant features in image data classification with help of CNN architecture with help of squeeze and excitation operators. Zheng et al.^15^ illustrated a solution for person re-identification with help of large pool of the image using pedestrian alignment network. System is tested with three type of datasets by deep learning network. The author proposed the method called attention guided CNN^16^ to detect thorax diseases. This inference techniques act as local and globally to diagnosis the thorax illness. Hence CNN is vital role in medical field. Irvin et al.^17^ focused on large radiograph dataset for chest radiographic studies.

Li et al.^18^ developed a new filter attribute selection technique for manifold learning based on the graph embedding architecture called as LLRFC score. However, the features chosen using this method may have a few redundancies. As a result, it is enhanced by removing attribute redundancy. LLRFC score + is the term given to the enhanced approach. Several alternative attribute selection methods are compared with author’s approaches on nine public tumor gene data. The experimental findings show the authors given technique is highly encouraging and applicable for tumor categorization.

Azzawi et al.^19^ designed a good approach for improving the prediction accuracy of Multi-Layer Perceptrons (MLP) neural networks by applying improved Particle Swarm Optimization (IMPSO). The IMPSO computes MLP weights and biases for more precise lung cancer prediction. This approach combines existing knowledge of lung cancer categorization based on gene expression data to improve classification accuracy. The cross-data set validations ensured the simulation’s dependability. Furthermore, when past knowledge was included, the result of the planned strategy improved.

Ludwig et al.^20^ focused a unique SR-based cancer categorization technique to support on gene expression data that considers all data's geometrical information. In other words, integrate the locally linear drive in technique into the sparse coding framework to protect the geometrical formation of all data. For result evaluation, the suggested method was used to six tumor gene expression datasets, demonstrating that it produces more classification accuracy than SR-based tumor categorization approaches.

Salem et al.^21,22^ provided a novel technique for categorizing human individual cancer illnesses based on gene expression profiles. The suggested method integrates Information Gain (IG) and the Standard Genetic Algorithm (SGA). It initially utilizes IG to pick features, then GA to reduce elements, and finally Genetic Programming (GP) to classify cancer types. Authors are evaluated this approach by identifying cancer illness in seven cancer datasets and comparing system performances to the mainly recent methods. The application of the proposed system to cancer datasets compared to other ML approaches demonstrates that no classification strategy consistently outperforms all others; nonetheless, GA improves the classification accuracy of different classifiers in general.

Yuana^23^ investigated the ML model using SVM and RF to detect the lung cancer. The authors addresses the difficultly in classifying lung cancer data with help of RF technique. Many researchers can use RF method in healthcare applications^24^.

Soni et al.^25^ endeavors to bridge the gap between genetic understanding and predictive modeling in lung cancer research. The hybridization of Convolutional Neural Network (CNN) shows a capable avenue for efficient and accurate classification of lung diseases, with potential implications for early detection and intervention. Hybridizing convolutional neural network model, emphasize the significance of contributions in advancing predictive modelling for lung diseases and address areas where further research is needed to enhance understanding and application in this critical domain.

Riaz et al. ^26^ introduces a robust framework for lung tumor image segmentation, combining the efficiency of MobileNetV2 with the adaptability of transfer learning. This method aligns with the evolving landscape of medical image analysis, offering a promising avenue for enhanced diagnostic capabilities and streamlined clinical workflows. The two-fold training involves initial segmentation refinement and subsequent fine-tuning, enhancing the model's capacity to capture intricate features. Results demonstrate the effectiveness of method, showcasing its potential for robust lung segmentation in chest X-ray analysis, contributing to improved diagnostic outcomes^27^.

Mazin Abed Mohammed et al.^28^ proposed an approach for multi-omics cancer detection within a scattered fog calculating model. Leveraging federated learning, they employ auto-encoders to locally process and encode multi-omics data at distributed fog nodes. This ensures data privacy and security. Subsequently, the encoded representations are aggregated at a central server. The fused information is then fed into an XGBoost classifier for cancer detection. This federated auto-encoder and XGBoost scheme not only enhances classification accuracy but also addresses the challenges of data decentralization and privacy concerns. By integrating multi-omics data and reinforcement learning, we contribute to a more holistic and adaptive framework for accurate cancer detection^29^.

In the previous work on LC prediction, MLP, random subspace, and SMO were employed. MLP in the previous work on LC prediction, MLP, random subspace, and SMO were employed. But large parameter counts can lead to overfitting, and training may be computationally intensive. Random subspace algorithm enhances model diversity by training on random subsets, reducing overfitting. However, limited interpretability and effectiveness depends on the quality of the random subsets. SMO is Suitable for large datasets, efficient in solving support vector machine optimization problems. At the same time, it can be sensitive to noise, and maintaining a single threshold may limit adaptability to complex data.

## Proposed system

This research addresses the critical issue of early prediction of lung cancer, the importance of primary finding and treatment for refining patient results. Proposed CNN used 5 × 5 convolution Layer, 2 × 2 Sub Sampling Layer, then 5 × 5 convolution Layer, 2 × 2 Sub Sampling Layer and 1 × 1 convolution Layer, 1 × 1 Sub Sampling Layer and applying, soft-max function to extract the normal and abnormal cells prediction.

### Weighted CNN based lung cancer prediction model

**Figure 1:**
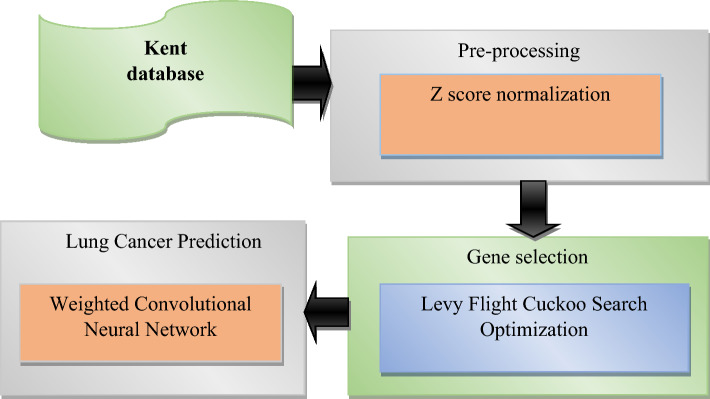
Architecture of the suggested technique.

**Figure 2:**
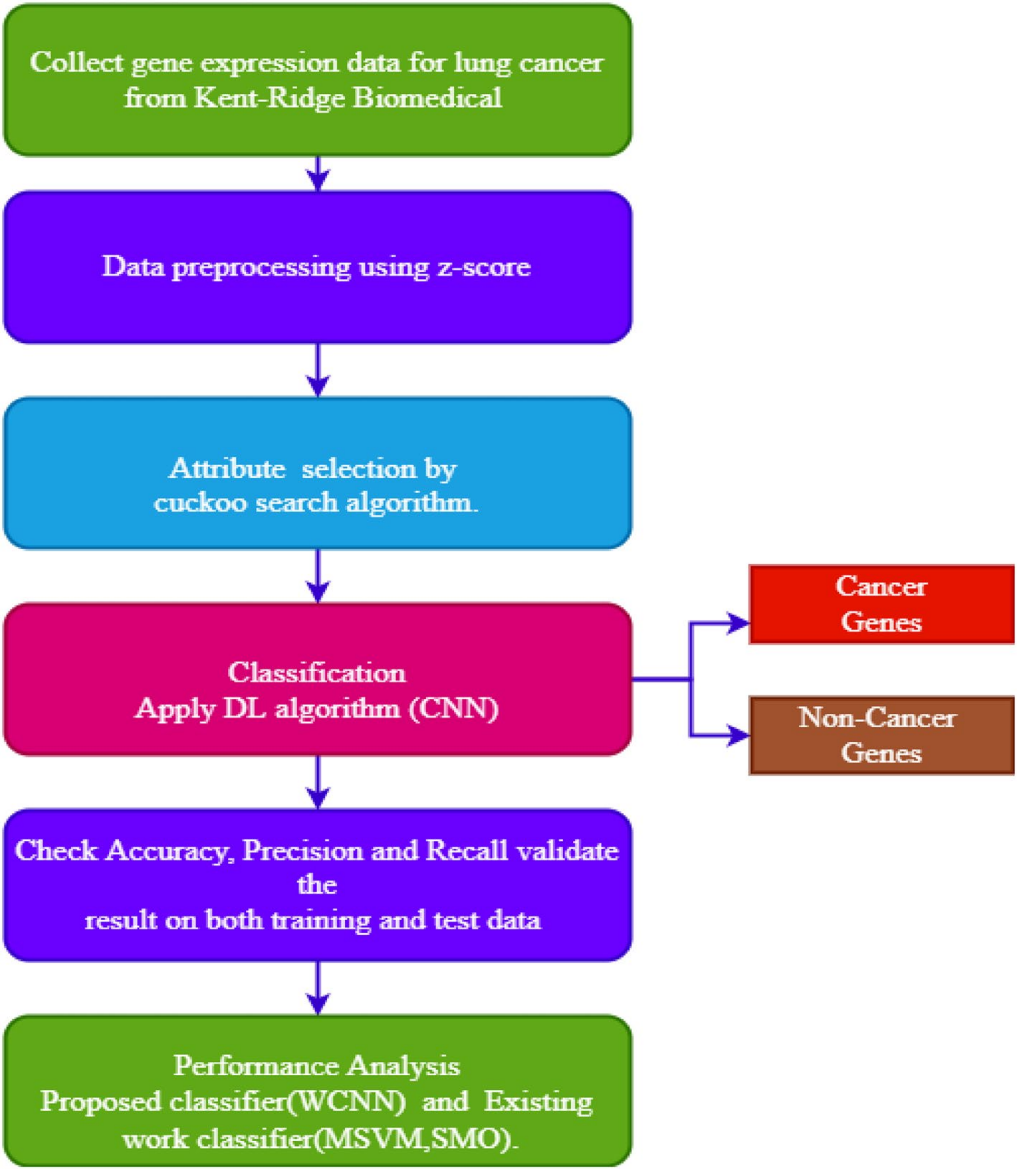
Lung cancer classification in genomic data by weighted CNN.

The proposed model utilizing Z score normalization, followed by significant gene selection using the LFCS algorithm. The last phase predicts the lung cancer. Figure 1 depicts the overall design of the suggested technique. The detailed view is represented in Fig. 2.

#### Input

Gene expression data for the lung cancer available in kent ridge biomedical repository. We can access /download the dataset by using the below link: https://leo.ugr.es/elvira/DBCRepository/. Data on gene expression are obtained for 86 main lung adenocarcinoma examples and 70 non-neoplastic lung tissues. There are 7129 genes in all of the samples. The entire dataset is separated into 70% as a training dataset and 30% as a test dataset and cross validation also performed for validating the outcome. The algorithm that produces the most reliable data on the test dataset is chosen as the best model.

#### Pre-processing using Z score normalization

Z-score normalization contributes to preprocessing by standardizing input values, ensuring a mean of 0 and a standard deviation of 1. Chosen for its simplicity and effectiveness, it enhances convergence and stability in the optimization process, improving the total result of the predictive model. The vital step for improving the result of ML approach is data pre-processing. First, normalization is performed to prevent data from being overburdened with one another. The normalization procedure converts data from disparate scales to the same scale. The mean and standard deviation of feature A is used to normalize values in this Z-score normalization procedure. The formula is as follows:

1 $$\text{v}^{\prime } = \frac{{ \text{v} - \bar{ \text{A}}}}{{\sigma _{ \text{A}} }}$$

in which, v′, v—new and old of each entry in data, respectively; σ_A_, ${\overline{\text{A}}}$—standard deviation and mean of A, respectively.

#### Gene selection using levy flight cuckoo search optimization algorithm

**Figure 3:**
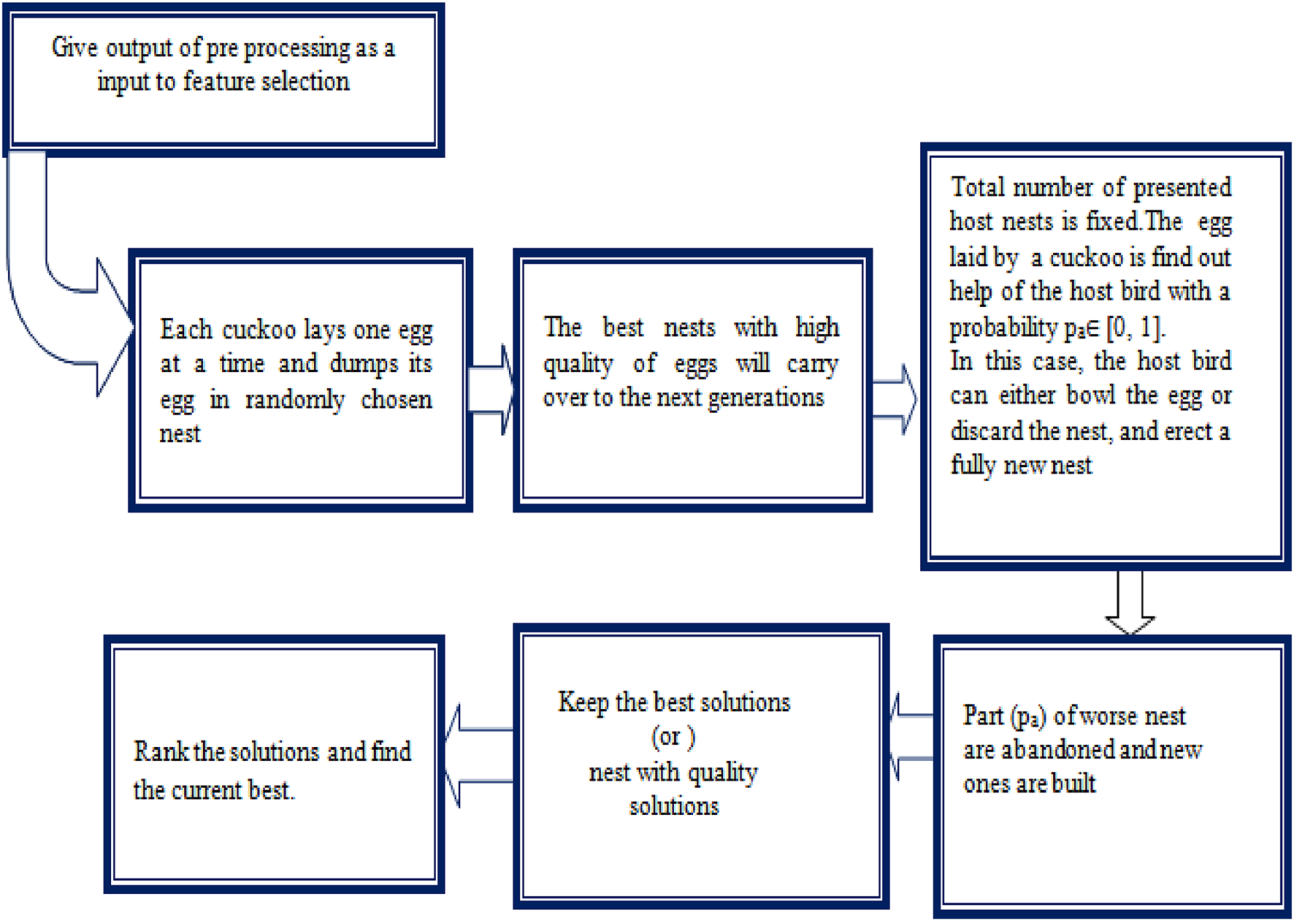
Algorithm flow.

After normalization, this scheme applied a gene selection technique based on the LFCS technique to reduce time consumption and increase the classification accuracy. Cuckoo Search (CS) is an innovative meta-heuristic model. This method was stimulated by some cuckoo species' makes brood parasitism, in which they put down their eggs in the nests of other host birds. Some host nests are capable of engaging in straight variance. The algorithm flow is indicated in Fig. 3.

If a swarm bird realizes that the eggs are not its own, it will either dispose of the foreign eggs or leave its nest and create a new one somewhere. In addition, other species have developed so that female freeloading cuckoos are frequently dedicated in the color and pattern imitation of a few selected host species' eggs^30–33^. This minimizes the likelihood of their eggs being discarded as a result, boosts their reproductive capacity.

The CS approach follows the following three romanticize rules:

1. Every cuckoo produces single egg at a time and places it in a nest selected at random;
2. The best nests with high-superiority eggs will be transferred down to future productions;
3. The amount of possible host nests is constant, and the egg leave by a cuckoo is recognized by the host bird with a likelihood is p_a_ ∈ [0, 1].

The fitness of a result might become the objective function for a gene selection issue (classification accuracy). Finally, every egg in a nest symbolizes an answer in this algorithm. A cuckoo egg indicates a recent solution; the goal is to use the effective and relatively improved clarifications (cuckoos) to substitute a not good solution in the nests^34–36^. The basic processes of the Cuckoo Search (CS) can be summarised using these above three principles.

The search for a new bird's nest place path is understand by the below Eq. (4).

2 $${\text{h}}_{{\text{j}}}^{{\left( {\text{t + 1}} \right)}} = {\text{h}} + \upalpha \oplus {\text{Levy}} \left(\uplambda \right);\quad {\text{j}} = 1,2, \ldots \ldots ..,{\text{n}}$$

${\text{h}}_{{\text{j}}}^{{\left( {\text{t + 1}} \right)}}$ represents i_th_ bird's nest position in the t production, α denotes step size control, α > 0; usually, α = 1 Levy (λ) is Levi’s search path randomly, it can express as follows by Eq. (5).

3 $${\text{Levy}}\,\left(\uplambda \right) = {\text{t}}^{{ -\uplambda }} ;\quad 1 <\uplambda < 3$$

The action size and identification chance of the CS approach are assigned to a constant value at the start and will not vary in consecutive stages in conventional cuckoo search. However, when the step size is exceeded, the search accuracy degrades, and it is straightforward to converge; when the size is smaller, the search speed decreases, and simple to slip into a local optimal. To overcome those issues in this work introduced an improved CS algorithm^37–39^.

The ICO algorithm integrates the action size with iterations, assigns a larger step extent at the start, and then reduces the step size as the iteration continues. The technique can attain global optimization, increase iterative speed, and improve search accuracy with a reduced step size^40–46^. The upgraded formula is

4 $$\propto_{{\text{i}}} = {\text{a}}_{{{\text{max}}}} \times \frac{1}{{\left( {\frac{{{\text{a}}_{\max } }}{{{\text{a}}_{{{\text{amin}}}} }}} \right)^{{\frac{{\text{t}}}{{\text{T}}}}} }} \times {\text{ran}}_{{\text{i}}} \times 0.01$$

a_max_, a_min_ represent the maximum and the least amount of step sizes, respectively. 'T' denotes the whole iterations^47^ a n_jd_ means the scope of the jth dimension of the dataset.

#### Algorithm for ICSO

**Table:** 

INPUT: lung cancer database
OUTPUT: Optimal genes
1: Create an initial population of N host nest x_j_∀j, j = 1… n
2: while t < Max Generation do
3: Obtain a cuckoo randomly by impose flights and calculate its fitness Fi
4: Select a nest j along with random N
5: if Fj > Fi then
6: Restore i by the fresh result for solution
7: end if
8: A portion (pa) of the bad nests is deserted, and new ones are constructed
9: Preserve the best results (solution with nest)
10: Rate the answers and determine the present best
11: while end

#### Lung cancer prediction using weighted CNN

Following gene selection, the data are categorized using a weighted CNN to identify normal and lung cancer cases. A CNN straightens the input to a vector. First, the layers of a CNN are chosen to fit the input data geographically. Then, CNN^48–51^ comprises one or more blocks of convolution and subsampling layers, followed by single or added completely connected layers and an output layer.

### Drawbacks of traditional CNN

The traditional CNN model employs a simple gene learning approach in the first layer, resulting in information loss. In this work, weighted CNN was used to solve this problem.

#### Weighted CNN

**Figure 4:**
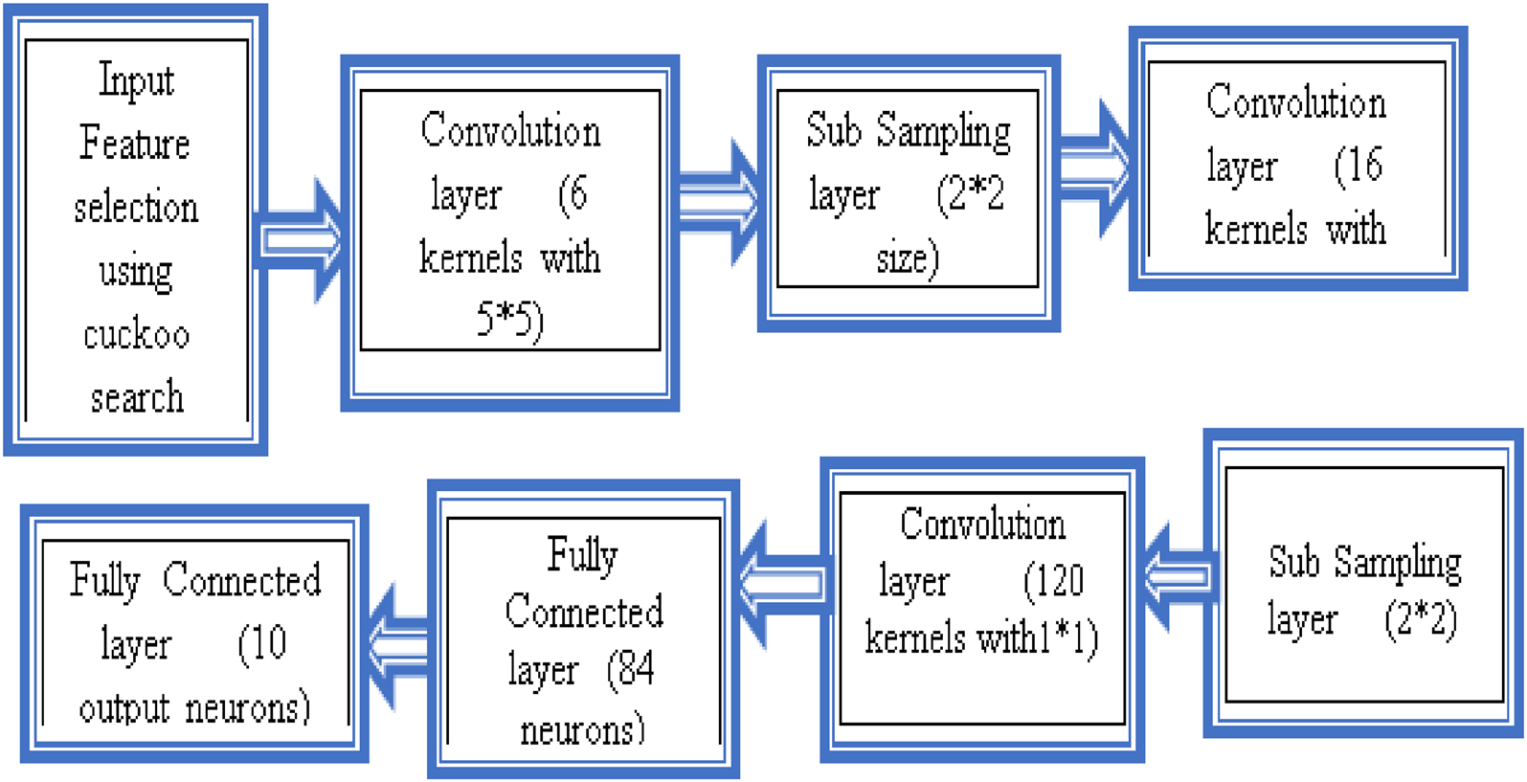
Layer views.

**Figure 5:**
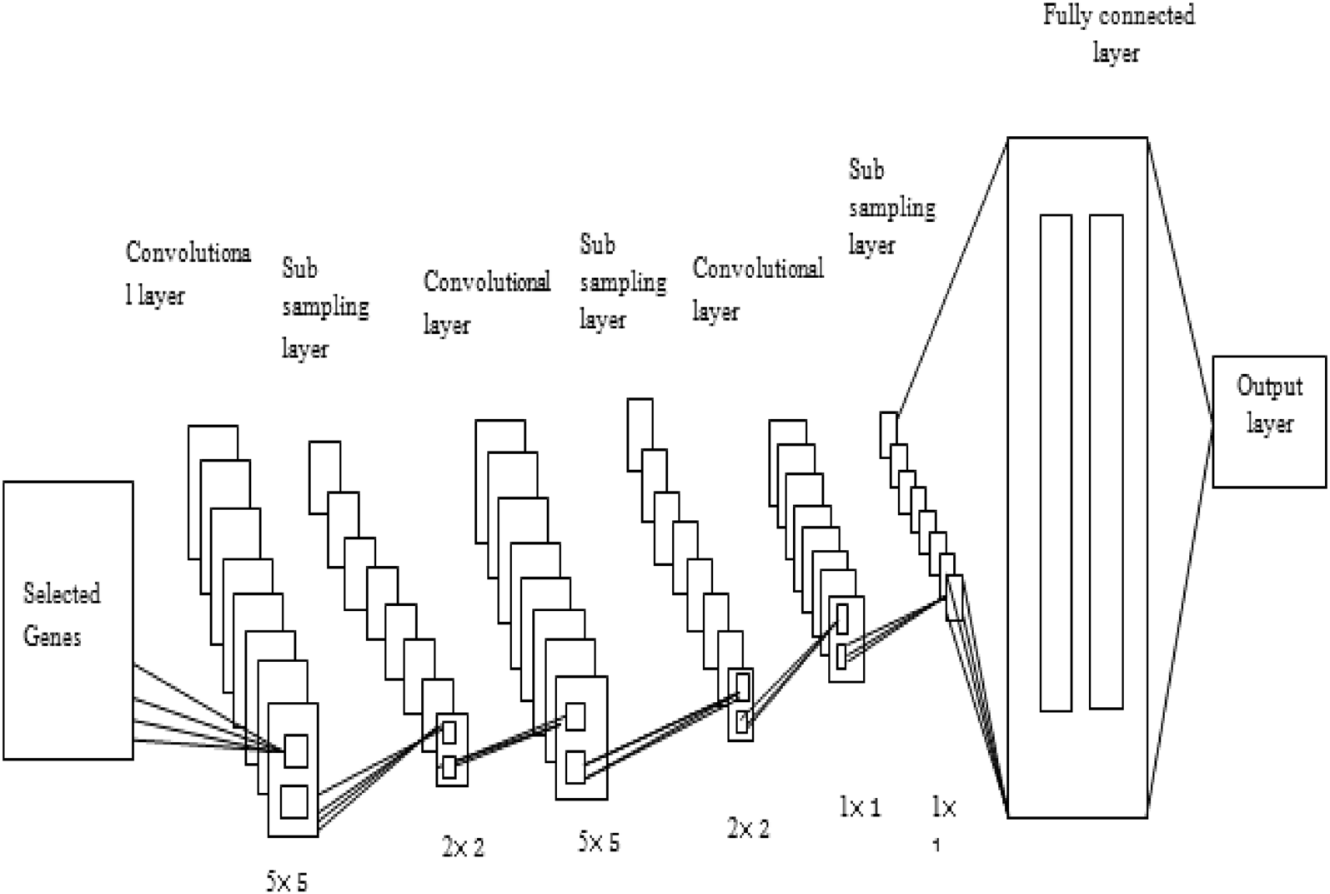
CNN architecture.

The CNN comprises four layers: a convolution layer (CL), a subsampling layer, a fully associated layer, and a production layer. The Layer views shown in the Figs. 4 and 5. The following sections provide a brief explanation of each sort of layer.

#### Convolution layer (CL)

Here, an input feature is combined with a kernel (filter) to produce n output feature maps. In general, a convolution matrix kernel is described as a pass through a filter, and the yield features acquired by combining the kernel and the key in are consigned to as attribute maps of size i*i. It has numerous CLs, and the attribute vector includes inputs and outputs of the subsequent CLs. Each CL contains lots of new n filters. These filters are combined with the input, and the depth of the resulting feature maps (n*) equals the number of filters used in the procedure. Each filter map is regarded as a discrete characteristic at a given point in the input side.

The result of the l-th CL is represented as ${\text{C}}_{{\text{i}}}^{{\left( {\text{l}} \right)}}$, comprises feature charactistic maps. It is evaluated as

5 $${\text{C}}_{{\text{i}}}^{{\left( {\text{l}} \right)}} = {\text{B}}_{{\text{i}}}^{{\left( {\text{l}} \right)}} + \mathop \sum \limits_{{{\text{j}} = 1}}^{{{\text{a}}_{{\text{i}}}^{{\left( {{\text{l}} - 1} \right)}} }} {\text{K}}_{{{\text{i}},{\text{j}}}}^{{\left( {{\text{l}} - 1} \right)}} {\text{*C}}_{{\text{j}}}^{{\left( {\text{l}} \right)}}$$

In which, ${\text{B}}_{{\text{i}}}^{{\left( {\text{l}} \right)}}$ represents bias matrix and ${\text{K}}_{{\text{i,j}}}^{{\left( {{\text{l}} - 1} \right)}}$ denotes convolution filter or function of size a*a links the j-th feature characteristic map in layer (l − 1) with the i-th feature characteristic map in a similar layer^21^.

X = conv wf (W, P) returns the convolution of a weight matrix W and an input P .

dim = conv wf ('size',S,R,FP) takes the layer dimension S, input dimension R, and function parameters, and returns the weight size.

dw = conv wf ('dw',W,P,X,FP) departures the derivative of X with respect to W.

6 $${\text{C}}_{{\text{i}}}^{{\left( {\text{w}} \right)}} = {\text{X}}$$

#### Subsampling or pooling layer

Its primary goal is to lower the magnitude of the feature maps derived from the earlier layer. The subsampling procedure is carried out between the mask and the feature maps. The most frequent pooling method is max pooling, in which the largest value of each block corresponds to the appropriate output feature.

#### Fully connected layer

It is a traditional feed-forward network with multiple hidden layers^52,53^. The output layer applies the Softmax activation function:

7 $$\begin{aligned} {\text{Y}}_{{\text{i}}}^{{\left( {\text{l}} \right)}} & = {\text{f}}\left( {{\text{x}}_{{\text{i}}}^{{\left( {\text{l}} \right)}} } \right), \\ {\text{Therefore}}\,{\text{x}}_{{\text{i}}}^{{\left( {\text{l}} \right)}} & = \mathop \sum \limits_{i = 1}^{{{\text{m}}_{{\text{i}}}^{{\left( {{\text{l}} - 1} \right)}} }} {\text{w}}_{{\text{i,j}}}^{{\left( {\text{l}} \right)}} {\text{y}}_{{\text{i}}}^{{\left( {{\text{l}} - 1} \right)}} \\ \end{aligned}$$

here, ${\text{w}}_{{{\text{i}},{\text{j}}}}^{{\left( {\text{l}} \right)}}$ denotes weights adjusted by this layer to create the illustration of every classes, and denotes the transfer function representing the non-linearity. The non-linearity in this layer is developed within its neurons.

#### Classification layer

It is usually the last layer in the network and is utilized to conclude. In addition, it defines how the network training hinders the difference between anticipated and accurate labels. Finally, the soft-max function is typically employed in the classification layer to get lung cancer prediction results.

A weighted Convolutional Neural Network (CNN) differs from traditional CNNs by assigning varying importance to different network elements. This weighting enhances adaptability, allowing the model to focus on crucial features. The impact on predictive performance is significant, as it enables targeted learning, emphasizing essential components. Weighted CNNs improve accuracy by capturing nuanced importance, leading to better generalization and robustness across diverse datasets, particularly in tasks like lung cancer prediction where certain features hold more significance. Computational challenges could include issues related to the complexity of the algorithm, dataset size, or computing resources.

Levy flight cuckoo search optimization, inspired by cuckoo bird behaviour, uses Levy flights for efficient search in gene space. It selects essential genes by adapting step lengths during exploration, optimizing gene subsets. Integrated with Z-score normalization, it enhances lung cancer prediction, addressing issues like MLP parameter redundancy.

In the context of lung cancer prediction, essential genes are determined based on their impact on predictive accuracy. The Levy flight cuckoo search optimization algorithm evaluates various gene combinations, assigning fitness values according to their contribution to the model's precision, recall, and accuracy. Genes that significantly enhance predictive performance are deemed essential. Integration with Z-score normalization ensures relevance to the overall dataset, promoting the selection of genes crucial for discriminating lung cancer patterns. The criteria prioritize genes that optimize the model's ability to distinguish between cancerous and non-cancerous samples.

## Experimental outcome

### Environmental setup

This part examines the outcomes of experiments conducted on proposed and current models. This model's implementation is carried out with the assistance of MATLAB. For the Kent Ridge Bio-Medical Dataset Repository, precision, recall, accuracy, and F-measure are evaluated to the existing variable SVM, RF, MSVM, SMO algorithm, and suggested WCNN.

### performance metrics

#### Accuracy measure

Accuracy is identified as the complete precision of the classifier model and calculated as the overall real parameter of the classification. It can understand by Eq. (8).

8 $${\text{Accuracy}} = \frac{{{\text{True}}\,{\text{Positive}}}}{{{\text{True}}\,{\text{Postive}} + {\text{False}}\,{\text{Positive}}}} \times 100$$

#### Precision and recall measure

Precision is computed with Predicted Positive Value (PPV) whereas Recall also named as sensitivity is computed by true positive rate is shown by Eq. (9) and Eq. (10).

9 $${\text{Precision}} = \frac{{{\text{True}}\,{\text{Postive}}}}{{{\text{True}}\,{\text{Postive}} + {\text{Fales}}\,{\text{Postive}}}}$$

10 $${\text{Recall}} = \frac{{{\text{True}}\,{\text{Postive}}}}{{{\text{True}}\,{\text{Postive}} + {\text{False}}\,{\text{Negative}}}}$$

#### F-Measure and error rate

F-measure is the test for positive class. It is the average of precision and recall of test. It is formalized as follows Eq. (11) and Error rate is represented by Eq.  (12).

11 $${\text{F - measure}} = 2 \times \frac{{{\text{Precision}} \times {\text{Recall}}}}{{{\text{Precision}} + {\text{Recall}}}} \times 100$$

12 $${\text{Error}}\,{\text{Rate}} = \frac{{{\text{Approximate}}\,{\text{value}}\,{\text{in}}\,{\text{the}}\,{\text{object}} - {\text{Exact}}\,{\text{value}}\,{\text{from}}\,{\text{the}}\,{\text{object}}}}{{{\text{Exact}}\,{\text{value}}}} \times 100$$

### Results and interpretation

Table 1 shows the performance of the WCNN validated using five folded cross validation matrix. The average performance of Accuracy, precision, recall and F-Measure are 85.02%, 86.35%, 85.57% and 85.95% respectively. The Table 2 shows the result of the existing methods and proposed WCNN method. From the data 70% of the dataset was taken for training and the remaining 30% for testing. The F-measure of proposed WCNN is 89.73%. The WCNN increases the F-measure and reduces the error rate as 10.89 percentage compared to existing system of SVM, RF, MSVM and SMO in without gene selection. Table 3 shows the result with gene selection. Here the F-measure of proposed WCNN is 92.45%. The WCNN increases the F-measure and reduces the error rate as 8.33% compared to existing system of SVM, RF, MSVM and SMO in without gene selection. Table 3 shows the result with gene selection. From the data 70% of the dataset was taken for training and the remaining 30% for testing. The Accuracy measure also increased in proposed WCNN compared to SVM, RF, MSVM and SMO in gene .selection and without gene selection.

**Table 1:** Performance of the WCNN validated using five folded cross validation matrix.

Iterations	Accuracy (%)	Precision (%)	Recall (%)	F-measure (%)
Iteration-1	84.30	86.20	85.51	85.85
Iteration-2	85.14	86.22	85.53	85.96
Iteration-3	85.15	86.41	85.66	85.98
Iteration-4	85.25	86.46	85.58	85.97
Iteration-5	85.28	86.48	85.59	85.98
Average performance	85.02	86.35	85.57	85.95

**Table 2:** Performance comparison results for different methods without gene selection.

Metrics	Methods				
	SVM (existing method)	RF (existing method)	MSVM (existing method)	SMO (existing method)	WCNN (proposed method)
Accuracy	78.93	79.25	81.12	85.45	89.10
Precision	78.32	79.57	81.78	84.14	90.28
Recall	77.40	78.13	80.19	85.11	89.18
F measure	77.85	78.84	81.43	84.67	89.73
Error rate	21.09	19.26	18.88	14.55	10.89

**Table 3:** Performance comparison results for different methods with gene selection.

Metrics	Methods				
	SVM (existing method)	RF (existing method)	MSVM (existing method)	SMO (existing method)	WCNN (proposed method)
Accuracy	78.93	81.52	84.46	87.12	91.66
Precision	78.65	80.25	80.85	88.78	93.01
Recall	77.72	78.00	79.43	86.65	91.91
F measure	78.18	79.10	79.01	87.34	92.45
Error rate	19.94	17.60	15.54	14.87	8.33

Table 4 shows the result without gene selection using 5-Fold cross validation. The accuracy measure increased in proposed WCNN compare to other techniques. Table 5 shows the result with gene selection using 5-Fold cross validation. The accuracy measure also increased in proposed WCNN compare to SVM, RF, MSVM and SMO techniques.

**Table 4:** Performance comparison results for different methods without gene selection using fivefold cross validation test.

Metrics	Methods				
	SVM (existing method)	RF (existing method)	MSVM (existing method)	SMO (existing method)	WCNN (proposed method)
Accuracy	76.30	76.94	78.10	82.53	85.02
Precision	76.44	76.89	78.87	81.10	86.35
Recall	75.86	75.40	77.18	82.89	85.57
F measure	76.91	76.64	78.01	81.67	85.95
Error rate	21.67	19.60	19.88	15.99	11.90

**Table 5:** Performance comparison results for different methods gene selection using fivefold cross validation test.

Metrics	Methods				
	SVM (existing method)	RF (existing method)	MSVM (existing method)	SMO (existing method)	WCNN (proposed method)
Accuracy	76.30	77.94	78.10	82.53	85.02
Precision	76.44	77.89	78.87	81.10	86.35
Recall	75.86	76.40	77.18	82.89	85.57
F measure	76.14	77.13	78.01	81.67	85.95
Error rate	20.42	19.24	19.54	15.99	11.90

**Figure 6:**
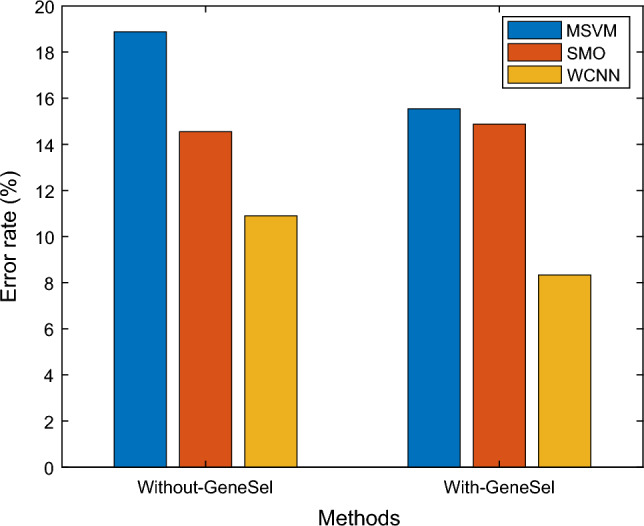
Error rate results from comparison of various classifiers.

The suggested WCNN's efficiency is demonstrated in the above Fig. 6 by contrasting it to the available SVM, RF, MSVM, and SMO approaches in terms of Error Rate. The proposed approach employs a feature selection stage, which reduces the result's error rate. Several techniques are depicted on the X-axis in the graph above, and Error Rate values are represented on the Y-axis. According to the outcomes, the newly introduced WCNN model provided Error Rate values of 8.33%, while the conventional MSVM and SMO methods produced only 15.54% and 14.87%, respectively.

**Figure 7:**
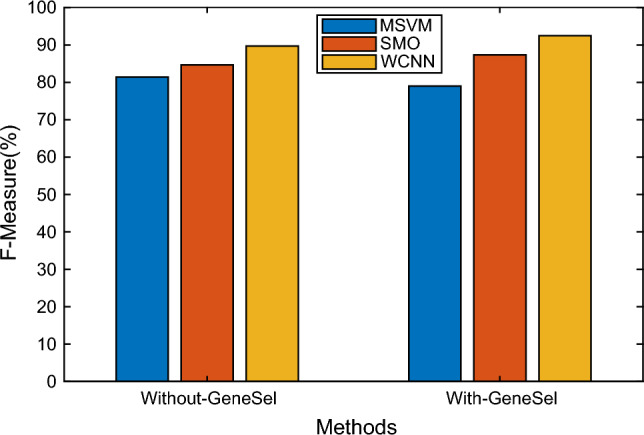
F-measure results versus classification methods.

Figure 7 indicate the result comparison of the existing classifier MSVM and SMO with the suggested WCNN algorithm in aspects of F-measure. Several approaches are depicted on the X-axis in the graph above, and F-measure assessments are represented on the Y-axis. According to the findings, the WCNN process offers higher F-measure values of 92.45%, while MSVM and SMO approaches yield only 79.01% and 87.34%, respectively.

**Figure 8:**
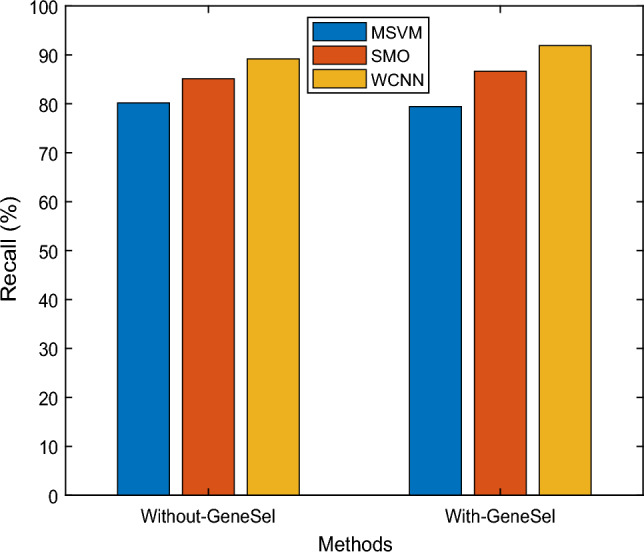
Recall results versus classification methods.

Figure 8 depicts a recall comparison between the existing MSVM, SMO, and suggested WCNN technique. The proposed approach employs a weight function in CNN for gene expression learning, which boosts the recall rate. Several systems are depicted on the X-axis in the graph above, and recall values are represented on the Y-axis. According to the outcomes, the WCNN method creates higher recall outcomes of 91.91%, while MSVM and SMO approaches yield only 79.43% and 86.65%, respectively.

**Figure 9:**
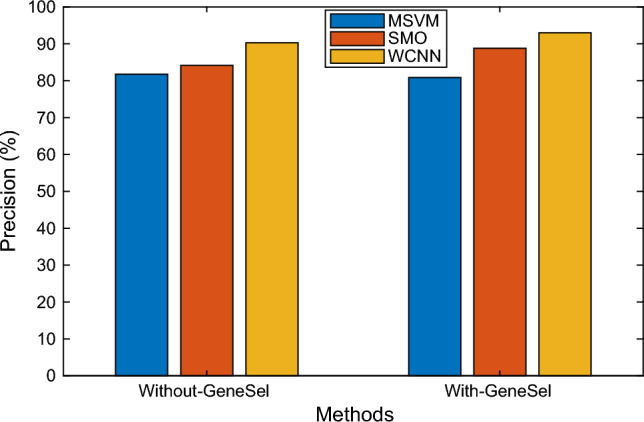
Precision results comparison of various classifiers.

Aspect of precision measure, the effectiveness of the WCNN is demonstrated in the Fig. 9 by comparing it to the existing MSVM, and SMO approaches. The proposed approach employs feature selection as a pre-processing step, which improves the result's precision. Several techniques are depicted on the X-axis in the graph above, and precision values are represented on the Y-axis. According to the findings, the WCNN system achieves precision results of 80.85%, while the existing HMM and FKNN techniques yielded only 88.78% and 93.01%, respectively.

**Figure 10:**
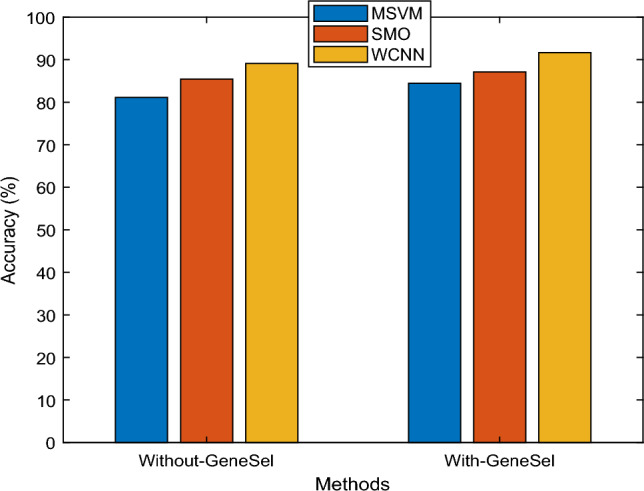
Accuracy results versus classification methods.

The above Fig. 10 chart depicts a performance comparison for Accuracy metrics with the existing classifier MSVM and SMO suggested WCNN algorithm. In the presented design, CS selects significant features using a probability function, which improves the accuracy of the WCNN. Several approaches are depicted on the X-axis in the graph above, and accuracy levels are represented on the Y-axis. According to the outcomes, the WCNN approach achieves better Accuracy results of 91.66%, while the existing MSVM and SMO techniques produced only 84.46% and 87.12%, respectively. The inclusion of error rate, indicating the proportion of incorrectly classified instances, contributes to a holistic understanding of the model's performance. By presenting this array of metrics, the study ensures a nuanced evaluation, addressing various dimensions of classification quality.

**Figure 11:**
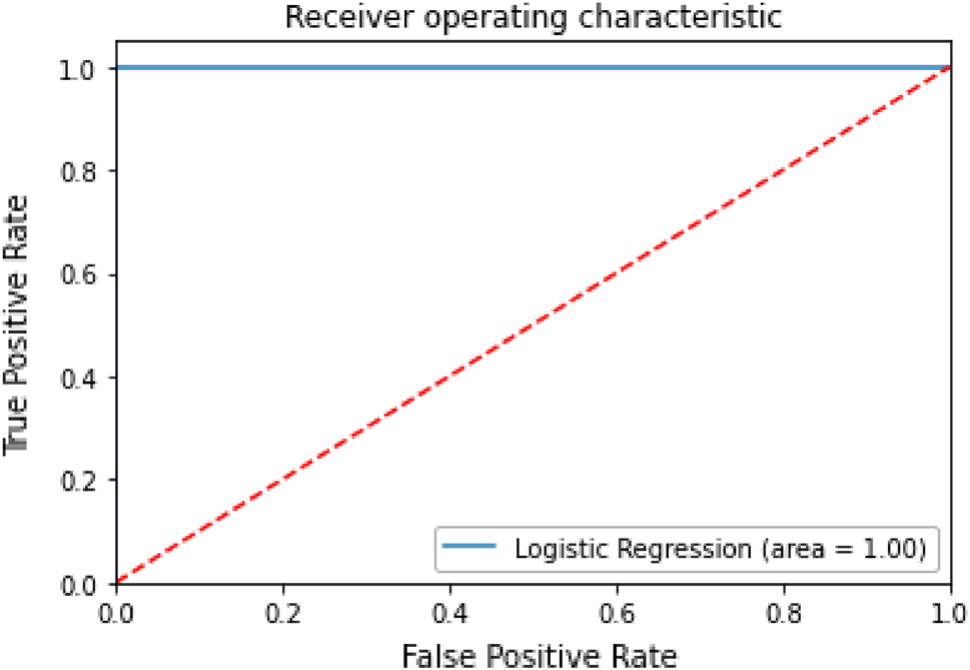
ROC curve.

Receiver Operating Characteristic curves helps to visualizing the result of a classification model. It represents the model efficiency finding true positives while avoiding the false positives. The Area Under Curve (AUC) value is shown in Fig. 11. It is value under 1.0. The 0.9–1.0 have excellent predictive ability.

**Psedo code to draw the roc curve**

Import roc_auc_score, roc_curve, plt from matplotlib.pyplot

Use roc_curve function to return fpr, tpr, thresholds

Plot([0,1],[0,1]

Set xlimt is 0.0 and 1.0

Set ylimit is 0.0, 1.05

Xlabel as (False Positive Rate)

Ylabel as (True Positive Rate)

Show the Log_Roc

## Practical advantage and limitation of the proposed research

The proposed technique offers practical advantages in lung cancer prediction. Firstly, by incorporating Z-score normalization, it ensures that gene expression data is standardized, improving the model's adaptability across diverse datasets. This standardization contributes to robust predictions, especially when dealing with variations in data distribution. Secondly, the integration of levy flight cuckoo search optimization addresses the challenge of attribute selection efficiently. This nature-inspired algorithm allows for an effective exploration of the gene space, enhancing the model's ability to select relevant features critical for lung cancer prediction. Additionally, the weighted Convolutional Neural Network provides a tailored approach by assigning varying importance to different elements, enabling the model to focus on crucial features and improving precision and recall. Overall, these practical advantages make the proposed technique not only effective but also adaptable to real-world scenarios, potentially offering advancements in early and accurate lung cancer diagnosis.

The proposed study has limitations. First, the efficacy on diverse datasets remains unexplored, affecting generalizability. Second, the computational demands of the novel technique could pose challenges for implementation in resource-constrained settings. Third, real-world clinical validation is crucial to ensure applicability.

## Conclusion

Cancer is main dangerous illness in the world. Therefore, cancer symptoms should be thoroughly researched before diagnosis to save patients' lives. As a result, an automatic prediction system for categorizing cancer based on gene expression data is required. This work aimed to provide an efficient mechanical model for lung cancer identification using gene expression data. First pre-processing will be performed using z score normalization to normalize the scale of the input values. And then, significant genes will be selected using the LFCS optimization algorithm. Finally, weighted CNN is employed for Lung Cancer Prediction. The findings show that this suggested technique model produces a better accuracy result which is 91.66%, than traditional models. The results suggest promising advancements in premature analysis and diagnosis of lung cancer, emphasizing the potential for practical implementation in clinical settings. However, deep learning makes higher computational complexities, so we need to use another model in the future.

Insightful future research suggestions.

- Conduct real-world clinical trials to validate the proposed technique's performance using patient data, considering diverse demographics and disease stages.
- Improve model interpretability to enhance trust by exploring methods for explaining and understanding the decision-making process.
- Assess the generalization capability of the technique across various datasets beyond the Kent Ridge Bio-Medical Dataset Repository.

## Data Availability

The datasets used during the current study are available from the corresponding author on reasonable request.
